# Loss of mitochondrial function induces transcriptomic signatures of Alzheimer's Diseases in mice

**DOI:** 10.1002/alz70855_101111

**Published:** 2025-12-23

**Authors:** Huanyao Gao, Kate Jensen, Jarred Nesbitt, Mark Ostroot, Priyanka Baloni, Cory C Funk, Jesse C Wiley, Jens O Watzlawik, Wolfdieter Springer, Alexander Galkin, Eugenia Trushina

**Affiliations:** ^1^ Yale University, New Haven, CT, USA; ^2^ Mayo Clinic, Rochester, MN, USA; ^3^ Department of Neurology, Mayo Clinic, Rochester, MN, USA; ^4^ Purdue University, West Lafayette, IN, USA; ^5^ Institute for Systems Biology, Seattle, WA, USA; ^6^ Sage Bionetworks, Seattle, WA, USA; ^7^ Mayo Clinic, Jacksonville, FL, USA; ^8^ Weill Cornell Medicine, New York, NY, USA; ^9^ Department of Molecular Pharmacology and Experimental Therapeutics, Mayo Clinic, Rochester, MN, USA

## Abstract

**Background:**

Early mechanisms of neuronal damage in Alzheimer's Disease (AD) include mitochondrial dysfunction and abnormal energy homeostasis. AD‐associated mitochondrial signaling was shown to occur upstream of amyloid precursor protein processing, Ab production, apolipoprotein E (APOE) expression, and tau pathology. However, whether alterations in mitochondrial function are sufficient to instigate AD remains unclear. Mitochondrial complex I (mtCI) is a rate‐limiting enzyme in the oxidative phosphorylation (OXPHOS) machinery. Changes in mtCI activity influence ATP and ROS production, cellular energy and redox homeostasis. We examined how reduced activity of mtCI due to a knockout of one of its accessory subunits *Ndufs4* in mice affects transcriptional networks in the brain. We also determined whether reduced activity of mtCI abrogates neuroprotective properties of a small molecule CP2.

**Method:**

RNA‐sequencing of the brain tissue was performed using *Ndufs4^‐/‐^* and control mice (*n* = 3–5 *per* group). The co‐expression modules of differentially expressed genes were compared to the AMP‐AD patient cohort data. Key mechanisms, including mitophagy signaling, were confirmed using ELISA and western blot analyses; the effect on mtCI activity was confirmed using functional assays.

**Results:**

In *Ndufs4^‐/‐^* mice, a residual activity of mtCI was ∼50% of the control. This loss of function led to a global disruption of mitochondrial homeostasis, energy metabolism, synaptic function, significant increase in mitophagy and a reduction in biogenesis. Transcriptomic signatures in brain tissue of male and female *Ndufs4^‐/‐^* mice overlapped with changer reported for AD patients and AD mouse models with Ab and *p*‐tau pathologies. These changes were partially rescued by a neuroprotective mitochondria‐targeting small molecule CP2. Consistent with studies in AD mice, CP2 treatment in *Ndufs4^‐/‐^* mice augmented the expression of genes associated with mitochondrial biogenesis and turnover, synaptic activity, autophagy, redox balance, and reduced expression of genes related to inflammation. Sex‐specific differences included better adaptation to a reduced activity of mtCI and to CP2 treatment in female mice.

**Conclusion:**

Reduced mtCI activity was sufficient to induce the AD‐like transcriptomic changes in brain. Residual mtCI function was sufficient to mediate neuroprotective effect of CP2, emphasizing a key role of mitochondria in AD development and treatment.

